# Vaccination is the most effective and best way to avoid the disease of COVID‐19

**DOI:** 10.1002/iid3.946

**Published:** 2023-08-08

**Authors:** Hadi Lotfi, Mina G. Mazar, Negar M. H. Ei, Mostafa Fahim, Nafiseh S. Yazdi

**Affiliations:** ^1^ Leishmaniasis Research Center Sabzevar University of Medical Sciences Sabzevar Iran; ^2^ Department of Medical Microbiology Sabzevar University of Medical Sciences Sabzevar Iran; ^3^ Department of Medical Laboratory Science Varastegan Institute for Medical Science Mashhad Iran; ^4^ Mashhad University of Medical Science Mashhad Iran; ^5^ Department of Pediatrics, Faculty of Medicine Sabzevar University of Medical Sciences Sabzevar Iran

**Keywords:** COVID‐19, vaccines

## Abstract

Most of the vaccines that are effective against SARS‐CoV‐2 have used the following functional strategies: inactivated viruses, live attenuated viruses, viral vector‐based vaccines, subunit vaccines, recombinant proteins, and DNA/RNA vaccines. Among the vaccines that stimulate the host's immune system with the help of DNA are: undergoing Phase 2/3 trials including INO‐4800 (International Vaccine Institute; Inovio Pharmaceuticals), Symvivo, Canada‐COVID19 (AnGes, Inc.); GX‐19 (Genexine, Inc.). BNT162b2 and mRNA‐1273 vaccines were made by BioNTech/Pfizer/Fosun Pharma group and Moderna/NIAID group, respectively, which are considered as types of RNA vaccines. Vaccines that are based on the viral vector are AstraZeneca, Sputonium, and Johnson‐Jensen. Among the inactive viral vaccines, the following can be mentioned: CoronaVac (Sinovac) WIBP vaccine (Wuhan Institute of Biological Products, Sinopharm), BBIBPCorV (Beijing Institute of Biological Products, Sinopharm), BBV152/Covaxin (Bharat Biotech, ICMR, National Institute of Virology) And among the protein‐based/subunit vaccines, the following can be counted: *NVX‐CoV2373*: (Novavax); SCB‐2019 vaccine (Clover Biopharmaceuticals AUS Pty Ltd.); *Covax‐19* (GeneCure Biotechnologies; Vaxine Pty Ltd.) mRNA vaccines, viral vector vaccines, and protein subunit vaccines cannot cause disease because these vaccines stimulate the immune system to produce antibodies against virus proteins instead of the virus itself (or its antigen). MRNA vaccines increase SARS‐CoV‐2 proteins and ultimately stimulate the production of T and B lymphocytes. The epidemic of HCoVs and their destructive and harmful effects on life has caused the scientific community to seek the production of an effective and efficient vaccine before its catastrophic release. We all need to know that none of us will be healed until the other is healed. The purpose of this review article is to present a selection of existing knowledge in the field of fighting and preventing the coronavirus.

## Graphical abstract

The safety of each of us depends on the safety of those around us. In addition to the importance of imposing public health and infection control measures to prevent or reduce the transmission of SARS‐CoV‐2, the most important step to contain this global pandemic is a vaccination to prevent SARS‐CoV‐2 infection in communities worldwide.

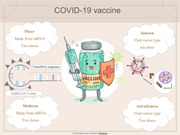

## 1 INTRODUCTION

### 1.1 Coronaviruses

Coronaviruses are RNA viruses belonging to the genus Orthocoronavirinae, in the family Coronaviridae, order Nidovirales. According to the information available so far, the genome of the coronavirus is the largest genome among the genomes of RNA viruses and is single‐stranded positive.

Orthocoronavirinae are divided into four subgroups^1^:

1. *Alphaacron (α‐CoV)*
2. *Beta‐coronavirus (β‐CoV)*
3. *Gama‐coronavirus (γ‐CoV)*
4. *Delta‐coronavirus (δ‐CoV)*.

SARS‐CoV‐2, which is a member of the β‐CoV subtype, reacts to heat and ultraviolet radiation. Based on this, it is found that high temperature has a significant effect on reducing virus replication.^2^ It is better to know that SARS‐CoV‐2 can withstand subzero temperatures. Like other respiratory pathogens, SARS‐CoV‐2 is transmitted through sneezing, coughing, or talking. People who are infected but show no symptoms make up 80% of the virus transmission.^3^, ^4^

In terms of genomic homogeneity, SARS‐CoV‐2 is 79.5%, 50%, and 87%–92% similar to SARS‐CoV, MERS‐CoV, and bats, respectively. M, N, and spike proteins (including S1 and S2) play an important role in all types of coronaviruses. The S1 subunit is primarily responsible for binding the virus to host receptors, whereas S2 integrates the virus with the host membrane.^5^

The mechanism of entry of viruses is similar, that is, by binding the receptor binding domain (RBD) (a component of the main protein, SPIRE protein, that the virus uses to bind to cells in the body) to functional receptors at the host cell surface.^6^

There is an angiotensin‐converting enzyme (ACE2), which is the most binding receptor for the virus and facilitates the entry of the virus. This host receptor has a higher affinity for SARS‐CoV‐2 than for SARS‐CoV.^7^

Pathophysiologically, the occurrence of cytokine storm and ARDS is the most important pathological symptom in all three viruses *MERS‐CoV*, *SARS‐CoV*, and *SARS‐CoV‐2*.^8^

In people with this disease, the cytokine release syndrome causes the disease to worsen due to the strong immune response in the body. These symptoms can lead to respiratory failure, lung tissue damage, and even death from cytokine storms.^9^

COVID‐19 was earlier considered as a respiratory and vascular viral disease because its causative agent (*SARS‐CoV‐2*) primarily targets the respiratory and vascular systems.^3^

SARS‐CoV‐2 is capable of causing disturbances in various organs such as the digestive system, cardiovascular system, hepatobiliary system, central nervous system, and kidneys, however, the respiratory system is the most affected by this virus. *SARS‐CoV‐2*‐induced organ dysfunction is generally explained by one or a combination of suggested mechanisms such as direct viral toxicity, ischemic injury due to vasculitis, thrombosis, immunoregulatory disorders, or renin impaired regulation of the angiotensin‐aldosterone system (RAAS).^3^

SARS‐CoV‐2 is the main cause of the spread of COVID‐19, which has become a pandemic. SARS CoV‐2‐SPL is composed of RBM (in the head position) and RBD (in the tail and trunk position) and is able to generate an effective response of B and T cells.

The genome of SARS‐CoV‐2 is more than 29 kb long and encodes four main structural proteins, spike (S), membrane (M), coat (E), and nucleocapsid (N) proteins.

The spike proteins are related to the entry and attachment of the virus and are the target of the neutralizing antibodies of the virus that no mutation has been observed near the second linker.

There is a part called RBD on spike virus protein that binds to ACE2. Antibodies made against RBD in SARS‐CoV cannot bind to SARS‐CoV‐2. SARS‐CoV antigens cannot be used to make a vaccine for SARS‐CoV‐2.

The RBD region was determined as Arg319 Phe541.

RMB is important for the production of neutralizing antibodies.

SARS‐CoV‐2‐SPL has 5 B‐cell epitopes, 15 CD4 T‐cell epitopes, and 17 CD8 T‐cell epitopes. The antigenicity of the S protein of SARS‐CoV‐2 has evolved and been captured by their novel antibody epitopes, which may lead to the direction of R and D activities during the development of vaccines.

The treatment of COVID‐19 depends on the clinical conditions and the condition of each patient. If the patient has mild symptoms, he does not need to be admitted to the hospital and it is better to treat him at home. In case of severe illness, hospitalization with treatment related to the patient's condition is essential In the current conditions, there are various treatments for the disease, including antiviral drugs (Molnupiravir and Remdesivir), and anti‐*SARS‐CoV‐2* monoclonal antibodies (Bamlanivimab/etesevimab, REGN‐COV2). Dexamethasone and immunomodulatory agents (e.g., Baricitinib, tocilizumab) are available under FDA‐approved emergency use (EUA) license or evaluation in the management of COVID‐19.^10^

Like other RNA viruses, *SARS‐CoV‐2*, while adapting to its new human hosts, is prone to genetic evolution by mutations over time, resulting in a variety of mutations that may have different characteristics from ancestral strains.^11^ During this epidemic, different types of SARS‐CoV‐2 were observed, and according to WHO, only a few of them had a significant impact on human health and life. As of December 11, 2021, five VOC *SARS‐CoV‐2* have been identified since the beginning of the epidemic, according to a recent epidemiological update by the WHO^3^, ^12^, ^13^:

Alpha (B.1.1.7): The first type of concern described in the United Kingdom in late December 2020,

Beta (B.1.351): First reported in South Africa in December 2020,

Gamma (P.1): First reported in early January 2021 in Brazil,

Delta (B.1.617.2): First reported in India in December 2020,

Omicron (B.1.1.529): First reported in Africa in November 2021,

Despite the unprecedented pace of vaccine development against COVID‐19 and strong global vaccination efforts including vaccine boosters, the emergence of these new *SARS‐CoV‐2* strains has undermined the significant progress made so far in limiting the spread of the disease.

Epidemiological data from several case studies have reported that patients with *SARS‐CoV‐2* infection have a live stool virus, suggesting possible fecal transmission through the mouth.^14^ A meta‐analysis of 936 infants from mothers with COVID‐19 showed that vertical transmission was possible but occurred in rare cases.^15^

People of different age groups are susceptible to this dangerous disease. However, patients over 60 years of age and patients with underlying medical conditions (obesity, cardiovascular disease, chronic kidney disease, diabetes, chronic lung disease, smoking, cancer, solid organ, or hematopoietic stem cell transplant patients) are at risk.^16^ Data shows that this disease is more severe in men and is associated with higher mortality.^17^, ^18^, ^19^ Also, ethnic and racial groups were hospitalized at a higher rate than whites. This high percentage of COVID‐19‐related hospitalizations among racial and ethnic groups was due to the higher risk of SARS‐CoV‐2 exposure and the increased risk of severe COVID‐19 disease.^3^

### 1.2 COVID‐19 vaccines

The most effective way to prevent viral diseases is vaccination. Viral vaccines reduce the severity of the disease in an individual and the transmission of pathogens to other susceptible individuals.^20^

Currently, the production and manufacture of vaccines is based on the production of a product with the ability to create strong, fast, long‐term immunity and protection, without side effects, which is modeled after the natural immune system. Therefore, vaccines aim to prevent the induction of effective molecules and cells that can eliminate the pathogen as quickly as possible.^6^ Operation warp speed (OWS) was a project aimed at the development, production, and distribution of faster and more effective vaccines and treatments and faster and better diagnosis of COVID‐19.

This partnership, which was started by the United States, predicted that due to the ineffectiveness or dangerousness of some vaccines, the plan would require a higher cost than the production of a normal vaccine, but one of the benefits of this plan was that the vaccine would be viable a few months earlier. It was available more than usual. The purpose of Operation Warp Speed was to create a broader effort in health and human services. The name of the project is taken from traveling faster than light and it started working with the aim of producing and delivering 300 million doses of effective vaccine by January 2021. As of August 2020, eight companies have been selected to receive approximately $11 billion in funding. The names of participants in the project, the various vaccine technologies, and which treatments received research funding are listed in Table 1.

**Table 1:** Types of COVID‐19 vaccines and their performance.

	Pfizer	Moderna	Janssen	AstraZeneca	Novavax
Number of doses	2, 21 days apart	2, 28 days apart	1	2, 4–12 weeks apart	2, 21 days apart
Approved for what ages?	+12 years	+18 years			
Effectiveness					
against death	100%				
Effective against					
current variants	Yes				
Effectiveness			72%	70%	
against disease in	95%	94.1%	86% against	100% for	95.6% (UK trials)
U.S. clinical trials	86% in 65+	86% in +65	Severe disease	Severe disease	
Emergency Use Authorization	December 11, 2020	December 18, 2020	February 27, 2021	Not Approved in the United States	Not Approved
Type of Vaccine	mRNA		Viral vector		Recombinant protein/ adjuvants
Most common	Fatigue, headache,				
Side effects	chills, muscle pain				
Testing for children?	Yes, ages 12–15	Yes, ages 12–17	TBD		
Who should not get the vaccine?	History of allergic reaction to polyethylene glycol, polysorbate or other vaccine ingredients Allergic reaction to first dose		History of severe reaction to vaccine ingredients, risk of rare blood clot in women under age 50	TBD	

Vaccines developed for COVID‐19 are divided into four categories^21^:

### 1.3 Nucleic acid vaccines

Nucleic acid vaccines in the form of DNA and RNA are used for COVID‐19. These vaccines give our body the genetic code necessary for our immune system to make the antigens needed to fight pathogens.^21^

With the help of genetic information in the form of deoxyribonucleic acid (DNA) or ribonucleic acid (RNA), these vaccines are able to produce proteins from SARS‐CoV‐2 that trigger the immune system's response. This design was proven for the first time in the COVID‐19 vaccine, and no licensed vaccine had this capability before.

DNA vaccines use bacterial plasmids encoded for a specific antigen that is able to produce protein with the help of this encoded fragment. On the other hand, in RNA vaccines, a messenger RNA (mRNA) or self‐amplifying RNA carries the antigen production message and causes the cell to make the target protein based on this message. The delivery of this RNA to cells can be alone, in encapsulated nanoparticles or with the help of some developed techniques (similar to DNA vaccines). Antigen production in a cell through DNA or RNA causes recognition and response by the immune system. This response includes killer T cells, antibody‐producing B cells, and helper T cells that support antibody production.^7^

Among the 12 licensed vaccines, only the results of phase 3 clinical trials of four vaccines that are undergoing phase 4 clinical trials are available. Two of the four candidates are mRNA‐based vaccines, while the other two are developed with adenovirus‐based nonreplicating viral vector technology.^22^

DNA vaccines contain information that is expressed as a protein antigen on the surface of the host cell and activates the host's immune system against SARS‐CoV‐2.

DNA vaccines are relatively easy to make and have fewer safety issues (compared to live vaccines).^23^

Viral vector vaccines, mRNA vaccines, and protein subunit vaccines carry messages for the immune system that cause antibodies to be made against the virus proteins, therefore these vaccines do not have the ability to cause disease in the case of the coronavirus. MRNA vaccines increase the production of SARS‐CoV‐2 proteins that stimulate the production of T and B lymphocytes by providing cellular material.^24^

Examples of vaccines made in this way: Pfizer and Moderna.^21^

#### 1.3.1 DNA vaccines

Examples: ongoing Phase 2/3 trials including INO‐4800 (International Vaccine Institute; Inovio Pharmaceuticals), GX‐19 (Genexine, Inc.) Symvivo; Canada—COVID‐19 (AnGes, Inc.).

Encoded plasmids have the ability to enter the nucleus of B cells, which is necessary for the production of S or RBD proteins. Among the advantages of this type of DNA vaccines, there is no need to move the live virus during preparation and the possibility of freeze‐drying, long‐term storage and transfer at room temperature (unlike ultra‐fragile mRNA vaccines). Although some plasmid‐derived DNA vaccines are in the final stages of testing and phase 3, they are still not licensed for general use. Currently, DNA vaccines are only approved for veterinary use.^25^

#### 1.3.2 RNA‐based vaccines (BNT162b2/mRNA‐1273)

The two approved RNA‐based vaccines are BNT162b2 developed by the BioNTech/Pfizer/Fosun Pharma group and mRNA‐1273 developed by the Moderna/NIAID group.^22^

mRNA molecules in RNA‐based vaccines are able to produce viral antigen proteins that stimulate the immune system. Lipid‐based complexes (e.g., liposomes) are used to prevent RNA degradation and increase efficiency and safe and correct drug delivery. Among the advantages of these vaccines are safe, tolerable and very compatible with new pathogens, ease of changing RNA formulas by artificially replacing some nucleosides with others (nucleoside‐modified mRNA is called Moderna). win. And one of the disadvantages of these vaccines is the production of interferon (caused by the presence of RNA), which causes the translation to take a long time and the vaccine is destroyed. Also, some RNA vaccines require two‐stage mass vaccination and extremely cold temperatures (−20 to −80 degrees Celsius) for storage and transportation, which makes their use impractical especially in developing countries.^8^

##### Vaccine: BNT162b2

###### Development

The fastest vaccine produced against COVID‐19 is Pfizer, which received emergency use authorization in December 2020. The phase I/II trial phase began in May 2020 and included 45 participants in three groups. Group 1 was divided into two doses of 10 μg for 21 days. Group 2 was given a dose of 30 micrograms at an interval of 21 days. Group 3 was given a dose of 100 micrograms.^26^

The part of the virus that binds to the host receptor and causes the virus to enter the cell is called the RBD. The IgG RBD is sufficient for serological tests because neutralizing antibodies can act here by preventing SARS‐CoV‐2 from entering people's cells.^26^

The conducted tests stated that the Pfizer vaccine is able to stimulate the immune system to produce antibodies against RBD SARS‐CoV‐2 and has acceptable safety and effectiveness.^26^ Forty‐three thousand four hundred and forty‐eight people over 16 years of age were selected for the vaccine test, 21,720 people received two doses of 30 μg for 21 days and 21,728 people received placebo, and finally, in November 2020, this vaccine completed its phase III trial with an effectiveness of 95.0%. On December 11, 2020, the FDA granted emergency use authorization for BNT162b28 and on August 23, 2021, the US FDA approved Pfizer's vaccine, making it the first approved COVID‐19 vaccine.^27^

###### The mechanism of action

Pfizer and Moderna vaccines are similar in terms of mechanisms of action and performance. The vaccine contains nucleoside‐modified mRNA encoding the spike glycoprotein of *SARS‐CoV‐2* and is delivered in lipid nanoparticles for more efficient delivery to host cells.^28^ The S2‐P antigen consists of the SARS‐CoV‐2 glycoprotein with a transmembrane anchor against which an antibody can be used to evaluate the effectiveness of the mRNA vaccine. The aim of the vaccine is to stimulate the reaction of the immune system against the spike protein. The data indicated that this vaccine is capable of generating antibodies against S2‐P and its RBD.^29^

###### Efficacy against multiple genetic strains of *SARS‐CoV‐2*

The first mutated case in the United States was reported on December 29, 2020, in Denver, Colorado. Two common mutant strains of SARS‐CoV‐2 were identified for the first time in the United Kingdom and South Africa. Since then, there have been numerous reports of several other mutant species around the world. These species have attracted public attention for two reasons^30^:

(a) Uncertainty about vaccine coverage against these mutations.
(b) These mutants have a higher reproduction ability.

Studies by Tian et al showed that the B.1.1.7 variant has a higher affinity for ACE2 binding and the reason for this higher affinity is the N501Y mutation. With the help of sera from patients vaccinated with BNT162b2 vaccine, the efficacy of this vaccine was evaluated against strains with N501Y mutation. As a result of this evaluation, no decrease in the effectiveness of the vaccine was observed. It is important to note that as mutations continue to be discovered, more research is needed to identify potential changes in vaccine coverage and dissemination rates.^31^

###### Efficacy of Pfizer vaccine against B.1.617.2

The first case of virulence of delta variant was in India in October 2020, which has a double mutation (two mutations in RBD). These mutations increase the capacity to bind to the receptor and transmit the virus. The effect of current vaccines on this variant is being investigated, however, in several countries, they reported a decrease in the effectiveness of the vaccine compared to the delta variant, so that the vaccine was not effective in preventing infection, but found it useful in reducing mortality and hospitalization. In England, the effectiveness against B.1.617 after the first dose was 33.5% and 59.8% after the second dose.^32^, ^33^, ^34^

###### Side effects and contraindications

The most common symptom that the participants showed after the injection was mild to moderate pain for 1 week. Injection site pain and systemic reactions in younger subjects (in case of pain 83.0% after the first dose and 78.0% after the second) than in older subjects (in case of pain 71.0% after the first dose and 66.0% after The second dose was more. Among the side effects, 39% of headaches in the elderly and 52% in young people, 51% of fatigue in the elderly and 59% in young people, 11% of fever in the elderly and 16% in younger people can be mentioned. It should be noted that 23% fatigue and 24% headache were reported in the participants who had injected placebo. Two anaphylactic responses were observed in two national health workers who received the BNT162b2 vaccine on the second day of the vaccine trial.^35^, ^36^

In adults, the occurrence of myocarditis after the injection of the vaccine was observed, and the FDA and CDC considered the effectiveness of the vaccine (due to the reduction of hospitalization and mortality) to be preferable to the increased risk of myocarditis. Bell's palsy is one of the side effects of viral vaccines, and in the case of the Pfizer vaccine ‐BioNTech has not seen a greater risk for this disease than other viral vaccines. Contraindications to the BNT162b2 vaccine, as specified in FDA guidelines, include those with severe allergic reactions to any component of the Pfizer‐BioNTech vaccine.^37^, ^38^

###### Management

The BNT162b2 vaccine is administered intramuscularly in the amount of 0.3 mL within 3 weeks. These vaccines should be stored on dry ice or in hospital freezers at very low temperatures. Vaccines can be used for up to 6 months if proper storage and transportation conditions are met. Vials can be thawed at room temperature (25°C) for up to 2 h or in a refrigerator (2–8°C) for up to 5 days.^37^

According to the FDA, the complete list of ingredients of the BNT162b2 vaccine includes mRNA, lipids, potassium chloride, potassium phosphate monobasic, sodium chloride, sodium phosphate dibasic dihydrate, and sucrose.^37^

##### Vaccine: MRNA‐1273

The results of another multicenter, phase 3, randomized, placebo‐controlled trial showed that subjects randomized to two doses of the vaccine (mRNA‐based mRNA‐1273, Moderna) 28 days apart had an immunity of 94.1%.^20^

Minor side effects have been reported including fatigue, muscle pain, headache, and pain at the injection site.^20^

mRNA‐based vaccines are highly potent and can be rapidly produced. The use of mRNA for vaccine production has several advantages, including the production of noninfectious vaccines with a reduced risk of mutation. Additionally, multiple mRNAs encoding multiple antigens can be delivered in a single vaccine. Additionally, multiple mRNAs encoding multiple antigens can be delivered in a single vaccine.^20^

###### Development

Moderna was one of the first companies that was active in the field of vaccine production and development against COVID‐19. On February 7, the first batch of Moderna vaccine was made, and on March 16, the first patient was injected with the mRNA‐1273 vaccine. Moderna was one of the first companies that was active in the field of vaccine production and development against COVID‐19. On February 7, the first batch of Moderna vaccine was made, and on March 16, the first patient was injected with the mRNA‐1273 vaccine. On May 1, Moderna began working with Lonza to produce one billion doses of mRNA‐1273. On May 12, the FDA granted mRNA‐1273 Fast Track designation as global demand for the vaccine increased. Following the successful results of phase 1 and 2 tests, on July 26, 2020, phase 3 tests were started. The analysis and analysis of the results indicated that the Moderna vaccine was more stable at refrigerator temperature, which had a positive effect on the transfer and distribution of vaccines. On November 30, 2020, Moderna applied to the US FDA for emergency use authorization and received approval shortly thereafter.^39^

On January 25, 2021, Moderna released the results of in vitro neutralization studies of sera from infected individuals, showing that the vaccine works against strains identified in the United Kingdom and the Republic of South Africa.^20^

###### The mechanism of action

The mode of operation and production of both Moderna and Pfizer vaccines is with the help of mRNA and is similar to each other.

###### Effect

Moderna vaccine mRNA‐1273 has an effectiveness rate of 94.1 after the second dose, but the effectiveness is less in people over 65 years old. The phase 3 COVE trial worked on groups at higher risk for the disease. Thirty thousand two hundred and forty participants were selected to evaluate the mRNA‐1273 vaccine and received control or two doses of the vaccine. Disease symptoms appeared in 185 people in the control group and 11 people in the vaccinated group. No serious side effects were observed in this vaccine.^20^, ^40^

###### Efficacy against multiple genetic strains of SARS‐CoV‐2

New cases of COVID‐19 disease caused by different strains in the United Kingdom (B.1.1.7 strain) and in South Africa (B.1.351 strain) have raised concerns about the effectiveness of vaccines. The evaluation of antibody activity in serum against B.1.1.7 and B.1.351 shows that the vaccine was effective against B.1.1.7 strain, however, in the case of B.1.351 strain, a decrease in antibody titer was observed.^41^

###### Effectiveness of moderna vaccine against B.1.617.2 (Delta)

Research on the response of the delta lineage of SARS‐CoV‐2 to the modern vaccine is limited, but a study from the Mayo Clinic Health System addressed this issue. This study, which was conducted between January and July 2021, saw an increase There were seven times more people with delta types in the geographic areas covered by the Mayo Clinic. This study, which has not yet been reviewed, shows that the effectiveness of the Moderna vaccine against delta is 76.0%.^42^

###### Side effects and contraindications

Moderna vaccine injection is associated with side effects such as pain, fever, fatigue, myalgia, arthralgia, nausea, vomiting, chills, erythema, swelling and lymphadenopathy. The percentage of local side effects at the injection site was very high and systematic side effects were observed in almost 50% of the subjects in the study after the second dose. However, these systemic side effects resolved within 2 days of receiving the vaccine.^40^, ^43^

###### Management

Moderna vaccine is injected into the deltoid muscle in two doses, in the amount of half a milliliter each time, with an interval of 28 days. Each vial contains 10 doses, which should be avoided from combining different vials or diluents into the vial. Vials should be transported at a temperature of −13 to 5 degrees Fahrenheit, but before opening, they should be stored for 30 days at a temperature of 36 to 46 degrees Fahrenheit and up to 12 h at room temperature between 46 and 77 degrees Fahrenheit. The components of this vaccine include mRNA, lipids, tromethamine, acetic acid, sodium acetate trihydrate, and sucrose.^43^

### 1.4 Vaccines based on viral vectors

This type of vaccine is produced by inserting the genome of the corona virus into a safe virus. The presence of this genome in a harmless virus cell stimulates the immune system and produces protein, which ultimately causes preparation against the corona virus. The virus used to carry it is weakened and cannot cause disease (carriers of viruses such as measles, vaccine, and adenovirus).^21^

Some viral vectors can replicate inside cells, but others cannot because the genes required for replication have been inactivated.^21^

Viral vector vaccines are also produced by the same rules. The viral vector carries antigen‐producing codes that stimulate the immune system. One of the disadvantages of these vaccines is the reduction of effectiveness when the host has been exposed to this vector. There are currently 16 nonreplicable vaccines and two candidate SARS vector vaccines. CoV‐2 is being developed clinically. Candidate *SARS‐CoV‐2* vector vaccines, all using nonreplicating viral vectors, have been approved by regulators worldwide for emergency use.^21^

Vaccines made this way: Ebola,, Sputonium,^21^ Oxford–AstraZenka: COVID‐19 vaccine (AZD1222). AstraZeneca and University of Oxford; Johnson and Johnson; Gamaleya (Sputnik V) Research Institute of Epidemiology and Microbiology, Ministry of Health of the Russian Federation. (Others under development: CanSino Biologics; Gam‐ COVID‐ Vac [Gamaleya Research Institute of Epidemiology and Microbiology, Ministry of Health of the Russian Federation]).^25^

The most common viral vectors are adenoviruses, retroviruses and vaccinia viruses. For example, the chimpanzee adenovirus known as ChAdOx1 has been used as the Oxford‐AstraZeneca vaccine vector. Among the advantages of this system, we can mention the proven experiences of this technology in gene therapy and its safety due to the lack of reproduction ability. In general, one of the disadvantages of these vaccines is that they may not be suitable for immunocompromised individuals as most may have been previously exposed to adenoviruses and may have prior immunity to them.^25^

Original antigenic sin, in the first contact with the virus, the body produces antibodies against the dominant antigen, and if in the future it encounters a more evolved type of virus that has another dominant antigen, it will still produce antibodies against the dominant antigen of the first encounter, as a result Antibodies made have no effect and immunity is reduced. This phenomenon is related to the development and implementation of SARS CoV‐2 vaccines. During research, CD4+ T cells that reacted with SARS‐CoV‐2 were found in people who were not infected with COVID‐19. Since these cells have a great affinity for HCoV‐OC43, HCoV‐HKU1 and HCoV α viruses, scientists believe that there is a possibility of cross‐reaction. The role of these T cells in the severity of the disease and the effect of this phenomenon on vaccination and pathogenicity are still unknown. To investigate the effect of corona vaccine on the antibodies against other viruses in the body, studies were conducted, and finally, the antibodies against the S1 protein of the α‐coronavirus HCoV‐229E and HCoV‐NL63 in the body did not increase, but antibodies which were against β‐coronaviruses HCoV‐OC43 and HCoV‐HKU1 showed an increase.

#### 1.4.1 Johnson & Johnson SARS‐CoV‐2 vaccine

##### Development

Johnson & Johnson spent more than a billion dollars to develop a vaccine with technology similar to the Ebola vaccine. In July 2020, they tested the safety and dose of the vaccine with people from the United States and Belgium. The third stage of vaccine evaluation was conducted with the participation of 44,325 people from the United States, Brazil and South Africa. Preliminary results to determine the effectiveness of the vaccine were released on February 9, 2021, and researchers are evaluating the prevalence of moderate to severe infections 14 and 28 days after vaccination.^44^, ^45^

The third approved vaccine for injection in the United States was Johnson and Johnson, which received an emergency use license after confirming the preliminary results of the third phase. As of May 2021, the Johnson & Johnson vaccine has been mass‐produced and doses are available to eligible individuals.^46^

One of the reasons why the Johnson & Johnson vaccine quickly gained global approval was its single dose and higher temperature tolerance, which was useful for rural communities and countries that do not have access to freezers. Bahrain was the next country to license its use on February 25, 2021, approving the vaccination for those 65 years of age and older and those with pre‐existing conditions.^20^

On September 7, 2020, the third phase of testing this vaccine to determine its effectiveness began with 44,000 participants. In February 2021, the US FDA granted emergency use authorization to Johnson & Johnson.^47^

### 1.5 The mechanism of action

To design the Johnson & Johnson vaccine, human adenovirus type 26 vector is used, which expresses the spike of SARS‐CoV‐2 and is responsible for cold symptoms. This vector is a type of natural virus, nonreplicating and noninfectious, which codes for the delivery of genetic material. It is used to spike antigen to human cells. After the delivery of this antigen, the immune system is stimulated and starts making antibodies against the spike so that the body can show resistance to the entry of the virus. An immune response is generated against the S antigen, leading to an antibody response to the vaccine against the spike protein in *SARS‐CoV‐2*.^38^

### 1.6 Effect

Currently, Johnson & Johnson's vaccine is in a Phase 3 trial called “Ensemble,” which will determine the effectiveness of a single‐dose regimen. Researchers are evaluating the effectiveness of the vaccine in preventing moderate to severe infections.^45^, ^48^

In the case of moderate infection, the effectiveness of the vaccine was described as 66.9% after 14 days and 66.1% after 28 days. In dealing with severe infection, 76.7% and 85.4% effectiveness was observed after 14 and 28 days after injection. Preliminary findings were released on January 5, 2021, and there were no hospitalizations or deaths related to COVID‐19 in participants receiving the Johnson & Johnson vaccine.^44^

### 1.7 Efficacy against multiple genetic strains of SARS‐CoV‐2

Unlike Moderna and Pfizer, Johnson & Johnson's Phase III trial examined the efficacy of the vaccine against new strains of SARS‐CoV‐2. The effectiveness of this vaccine on “501Y.V2” strain After 14 days of injection, 52% of people with moderate infection and 64% of people with severe infection were reported. Also, the effectiveness of the vaccine after 28 days of injection was described as 73.1% in people with moderate infection and 81.7% in people with severe infection. The investigations carried out for the selection of a vaccine in South Africa were very important because a high‐efficiency vaccine was needed to fight the 501Y.V2 strain. In terms of vaccine‐neutralizing antibodies and immunogenicity, early studies by Johnson and Johnson showed a 1.6‐fold decrease in delta‐neutralizing antibodies however, there is still strong evidence that the vaccine can prevent hospitalization and prevent death (the most important goals of vaccination).^20^

### 1.8 Safety profile: Side effects and contraindications

Only people who were severely allergic to Johnson and Johnson vaccine components were not allowed to use this vaccine.^49^

During the trials, the complications caused by this vaccine were excessive response to the vaccine, swelling of the face, pain at the injection site, and deep vein thrombosis, which was shown by further investigations that the deep vein thrombosis was not caused by the injection of the vaccine.^50^

Pain at the injection site, erythema, swelling, and systemic reactions such as headache, fever, fatigue, and myalgia were among the side effects that were mild in most of the participants and less than 1% of people caused disruption in daily functioning or hospitalization. The FDA has published changes to the EUA Fact Sheet warning of the potential but rare risk of Guillain‐Barré syndrome (GBS) and thrombosis associated with thrombocytopenia (TTS).^51^

The CDC found that the benefits of the vaccine in preventing hospitalization and death far outweighed the potential risk of TTS or GBS.^51^

From April 13, 2021 to April 23, according to the opinion of FDA and CD, the use of Johnson and Johnson vaccine to investigate the effect of this vaccine on thrombosis, blood disorders, and other complications was prohibited. The reason for this was the observation of six cases of cerebral sinus thrombosis (CVST) in women aged 18–48 years immunized with Johnson & Johnson vaccine. The FDA and CDC have concluded that the vaccine is safe and effective and therefore, the risk of developing CVST and thrombotic thrombocytopenia syndrome is very small compared to the benefits of the vaccine.^52^

### 1.9 Management

One of the advantages of this vaccine compared to other vaccines is its single dose (0.5 mL intramuscular injection) and storage at a higher temperature. Each vial contains five doses, which can be stored for up to 6 h at 36–46 degrees Fahrenheit if punctured, or up to 2 h at room temperature of 77 degrees Fahrenheit, and up to 12 h at 77–47 degrees Fahrenheit if not punctured. The ingredients of this vaccine are citric acid monohydrate, trisodium citrate dihydrate, ethanol, 2‐hydroxypropyl‐beta‐cyclodextrin, polysorbate‐80, and sodium chloride.^20^

#### 1.9.1 ChAdOx1 nCoV‐19 vaccine

The next approved vaccine is a nonreplicating virus vector. Vaxzevria was developed by Astrazenka and the University of Oxford by modifying an adenovirus called ChAdOx1‐S, which shows a range of 62‐90% in phase III. ChAdOx1‐S is a simian adenovirus vector vaccine that contains information for the expression of the *SARS‐CoV‐2* spike protein.^22^

During the conducted tests, the efficacy and immunogenicity of the vaccine was evaluated as 64% after the first dose and 70.4% after the second dose. ChAdOx1 nCoV‐19 vaccine has been approved or licensed for emergency use in the prevention of COVID‐19 in many countries around the world but has not yet received EU or FDA approval for use in the United States.^3^

Viral vaccines have the ability to stimulate the immune system without adjuvants. In this type of vaccines, viral antigens are used to stimulate the immune system. Recombinant adenovirus vectors are relatively safe and their use can elicit strong and widespread humoral and cellular immune responses.^23^

#### 1.9.2 Gam‐COVID‐Vac/Sputnik V

The Gam‐COVID‐Vac/Sputnik V vaccine was developed at the Gamaleya Research Institute in Russia with the help of viral vector technology that carried the glycoprotein S gene. In this strategy, Ad26 is used as “prim” and Ad5 as “boost.” The main advantage of this type of vaccine is the stronger and longer‐lasting immune response that can be achieved with the primary heterologous booster approach. Data from almost 22,000 participants reported that Gam‐COVIDVac was 92% effective, establishing itself as one of the most effective vaccines.^22^

#### 1.9.3 Ad5‐nCoV and Ad26.COV2. S

Ad5‐nCoV and Ad26.COV2 are both viral vector vaccines that encode sequences related to the S protein. Ad5‐nCoV was developed by CanSino Biological Inc. and Ad26.COV2 was developed by Janssen Pharmaceuticals. Both vaccines are single dose and do not need to be stored in the freezer, which is one of their advantages. Therefore, if further data on this vaccine candidate is encouraging, single‐dose administration could facilitate rapid and efficient mass vaccination.^22^

### 1.10 Viral vaccines

Consists of two types of vaccines: 1‐ Live attenuated 2‐ Inactive.^21^

Live attenuated vaccines use a weakened virus that grows and replicates but does not cause disease. These vaccines include oral measles, yellow fever, and polio. In this type of vaccines, to be safe, the genetic material of the virus is destroyed by heat, radiation, etc and cannot reproduce but can stimulate an immune response. Some of these vaccines include seasonal flu and polio.^21^

The live attenuated *SARS Covid 2* vaccine stimulates mucosal and cellular immunity but may be found in feces, thereby causing transmission. It is also possible to recombine. Finally, making this vaccine is difficult and requires intensive work.^21^

#### 1.10.1 Inactivated virus vaccines

An inactivated viral vaccine stimulates the immune system with the help of an inactivated sample of the pathogenic virus and creates a strong response, but is not capable of causing disease. Several types of vaccines were produced with this strategy and received the license for use. These vaccines include^22^:

These are the CoronaVac (Sinovac) WIBP vaccine (Wuhan Institute of Biological Products, Sinopharm), BBIBPCorV (Beijing Institute of Biological Products, Sinopharm), BBV152/Covaxin (Bharat Biotech, ICMR, National Institute of Virology).^22^

Sinovac Biotech (Sinovac Research and Development Co., Ltd./Butantan Institute); Sinopharm; Bharath Biotech.^22^

CoronaVac and WIBP vaccines are SARS‐CoV‐2 virus strains that have lost their pathogenic power with beta‐propiolactone. During various tests, the production of antibodies against 10 strains of SARS‐CoV‐2 was proven by CoronaVac. Likewise, in the case of the WIBP vaccine, antibodies were produced in the body after receiving a single dose, and no side effects were seen. The BBIBP‐CorV vaccine made by Sinofarm is very similar to the previous vaccines and differs only in the virus strain used. This vaccine also showed positive results during tests, and due to the presence of a complete virus, it is able to create different but weaker immune responses in the body.^22^

### 1.11 Protein/subunit‐based vaccines

These types of vaccines do not contain genome and contain the proteins of the corona virus in full or part of the protein or based on nanoparticles. Vaccines that contain protein fragments are made using the recombinant protein technique, which is easier to produce. These types of vaccines include whooping cough, streptococcus pneumonia, and so on. These vaccines contain the proteins of the coronavirus that the immune system recognizes and creates. Corona protein vaccine creates a high level of immunity and needs to strengthen the immune response.^21^

Protein‐based vaccines include subunit vaccines and virus‐like particles. Protein subunit vaccines are easily produced and are more tolerable in people, but they are less immunogenic, so adjuvants are used to solve this problem. In this vaccine, recombinant proteins and antigenic parts of the virus are used for immunogenicity. Currently, 33 candidate SARS‐CoV‐2 protein subunit vaccines are in clinical development, of which at least one has been shown in phase III clinical trials to induce high titers of neutralizing antibodies.^21^

In a group of protein vaccines, the empty shell of the virus is used to deceive the immune system. These types of vaccines are not pathogenic because they do not contain genome. Available vaccines are similar to human papillomavirus vaccines.^21^

Since protein subunit vaccines are produced from viral fragments, they have the ability to stimulate the immune system. For each of these vaccines, antibodies against *SARS‐CoV‐2* proteins circulate after vaccination to fight future infections.^22^

Examples: NVX‐CoV2373: (Novavax); SCB‐2019 vaccine (Clover Biopharmaceuticals AUS Pty Ltd.); Covax‐19 (GeneCure Biotechnologies; Vaccine Pty Ltd.).^53^

The participating antigens in subunit vaccines that have the ability to create immunity are usually S or RBD proteins and are known as SARS‐CoV‐2 immune protection. However, one of the disadvantages is that they often require an effective adjuvant to achieve a stronger immune response and, in some cases, adjuvants may cause strong allergic reactions to vaccines.^53^

#### 1.11.1 Epi Vac Corona

The manufacturer of EpiVacCorona is Vector Institute, and it was used without further testing and only by testing it on 100 people in Russia. To date, this is the only vaccine composed of *SARS‐CoV‐2* antigenic peptides that have been approved for use, despite no published results in the medical literature.^22^

#### 1.11.2 ZF2001

Among other vaccines is ZF2001 protein subunit which uses receptor‐binding domain as an immune system stimulant and has shown good results in the first and second phase of the trial. These trials support the use of a three‐dose schedule that will be further tested in a Phase III trial to broadly evaluate the safety, immunogenicity, and efficacy of the ZF2001 vaccine.^22^

#### 1.11.3 Genrex biotechnology peptide vaccines

Genres Biotechnology uses a direct peptide approach. The company has developed a peptide vaccine against *SARS‐CoV‐2* using synthetic viral peptides as immunogens and using a proprietary Ii‐KeyÔ immune activation platform.^23^

#### 1.11.4 NVX‐CoV2373 vaccine

This vaccine was produced by Novavax company, NVX‐CoV2373 for the prevention of COVID‐19 disease. It is a stable pre‐fusion protein incorporated into the company's proprietary nanoparticle platform (Matrix‐MÔ) to enhance immune responses and stimulate higher concentrations of neutralizing antibodies in the blood.^23^

The results of the phase 2 trial in South Africa confirmed the effectiveness of the NVX‐CoV2373 vaccine. The trial was conducted as the country faced a second wave of infections caused by the beta (B.1.351) strain, which shows its effectiveness against the virus.^3^

## 2 CONCLUSION

The safety of each of us depends on the safety of those around us (Table 1).^54^

To control the disease and reduce or prevent the transmission of Sars‐COV‐2 disease, it is necessary to vaccinate all people in addition to observing personal and public health tips. The efforts of the researchers and researchers made the vaccine effective at the virus at high speed and cause the devastating virus to make the virus. Vaccination stimulates the immune system and leads to the production of neutralizing antibodies against *SARS‐CoV‐2*.^3^

Advances in the field of vaccine production give us hope for the production of heat‐stable vaccines. Such vaccines can be used in tropical environments without the necessary resources. Countries around the world, regardless of political ideologies, can unite and work together to quickly and successfully roll out a COVID‐19 vaccine worldwide.^7^

Vaccine manufacturing companies use different strategies, but the most used strategy in the production of the COVID‐19 vaccine was to introduce an antigen into the body to stimulate the immune system. This antigen can be a nonpathogenic protein or an inactive viral particle. A group of vaccines also work by transferring mRNA related to viral proteins, which produces this protein in the body, causing the immune system to react (Pfizer‐BioNTech and Moderna). Another group of vaccines use a modified viral vector to deliver the virus protein and activate the immune system (AstraZeneca‐Oxford and Janssen's Johnson and Johnson). Viral vector for the AstraZeneca‐Oxford chimpanzee adenovirus vaccine and for the Janssen's Johnson human adenovirus vaccine.26 that both of these carriers are not capable of reproduction. Among the vaccines produced in the subgroup are Novavax and GSK‐Sanofi protein subunit vaccines, which require adjuvants for better performance. In the process of making these vaccines, we need baculoviruses to produce recombinant proteins in insects. The protein subunit approach has been used to develop other common vaccines (e.g., influenza, hepatitis B, and human papillomavirus vaccines).^24^

More studies are needed to determine the effectiveness of each vaccine and the number of doses required to have an effect on different strains of SARS‐CoV‐2. Pfizer and Moderna vaccines use a similar mechanism for immunogenicity. In addition, the effectiveness of both has been evaluated at almost 95%, but the Johnson and Johnson vaccine has a different mechanism (using a viral vector to strengthen the immune system) and is less effective in preventing the disease. Each vaccine has very high effectiveness in preventing hospitalization and death, which are the most important goals of vaccination.^20^

One of the complications of ChAdOx1 nCoV‐19 and Ad26 vaccines is thrombosis with thrombocytopenia in different locations (cerebral venous sinus thrombosis/splanchnic venous thrombosis), which was called vaccine‐induced immune thrombocytopenia (VITT). Prevention, control, and treatment of VITT are similar to HIT.^3^

Different countries are planning to inject the third dose of vaccine for their people. Studies show that after two vaccine doses, there is a slight decrease in immunity and the third dose provides higher levels of protection.^3^

A phase 2 randomized controlled trial from the United Kingdom comparing different combinations of booster regimens concluded that mixing vaccine types improved antibody as well as neutralizing responses for all seven vaccines studied, which included most of the major commercially available vaccines.^20^ The rapid response of pharmaceutical companies to the COVID‐19 pandemic cannot be understated and with vaccine distribution ramping up globally, the fight against a virus that has killed nearly millions appears to be finally tipping in humanity's favor.^5^

## AUTHOR CONTRIBUTIONS

**Hadi Lotfi, Mina G. Mazar, and Nafiseh S. Yazdi**: Wrote the first draft of the manuscript. **Mostafa Fahim and Negar M. H. Ei**: Contributed to revise the article critically for important intellectual content. **Hadi Lotfi, Mina G. Mazar, and Nafiseh S. Yazdi**: Collaboratively contributed to the concept of the original research resulted in this report. All authors reviewed and approved the final version of the manuscript. The authors contributed equally to all aspects of the article.

## CONFLICT OF INTEREST STATEMENT

The authors declare no conflict of interest.

## Data Availability

The data that support the findings of this study are available from the corresponding author upon reasonable request.
